# Habitat connectivity is determined by the scale of habitat loss and dispersal strategy

**DOI:** 10.1002/ece3.4072

**Published:** 2018-05-17

**Authors:** Allan H. Edelsparre, Ashif Shahid, Mark J. Fitzpatrick

**Affiliations:** ^1^ Department of Biological Sciences Integrative Behaviour and Neuroscience Group University of Toronto Scarborough Toronto Ontario; ^2^ Department of Ecology and Evolutionary Biology University of Toronto Toronto Ontario; ^3^ Department of Cell and Systems Biology University of Toronto Toronto Ontario

**Keywords:** behavior genetics, connectivity, dispersal, habitat loss, landscape ecology, threshold

## Abstract

Understanding factors that ameliorate the impact of habitat loss is a major focus of conservation research. One key factor influencing species persistence and evolution is the ability to disperse across increasingly patchy landscapes. Here we ask whether interpatch distance (a proxy for habitat loss) and dispersal strategy can interact to form thresholds where connectivity breaks down. We assayed dispersal across a range of interpatch distances in fruit flies carrying allelic variants of a gene known to underlie differences in dispersal strategy. Dispersal‐limited flies experienced a distinct negative threshold in connectivity at greater interpatch distances, and this was not observed in more dispersive flies. Consequently, this differential response of dispersal‐limited and more dispersive flies to decreasing connectivity suggests that habitat loss could have important implications on the evolution and maintenance of genetic variation underlying dispersal strategy.

## 1 INTRODUCTION

Understanding how human activities shape the evolutionary dynamics of biodiversity represents one of the biggest challenges of our time. Life on earth is being particularly challenged by human impact leading to habitat loss and fragmentation. This includes threats to biodiversity from development, roads, and land clearances such as logging and agriculture (Isbell, Tilman, Polasky, & Loreau, 2015). Concerns are driven by uncertainty as to how these activities ultimately affect ecosystem function and services (Loreau, 1998). Habitat loss influences the availability of resources, and this can have important implications for the competitive interactions within communities by removing important refuges for prey (Dytham, 1994), altering the dynamics of migratory networks (Betini, Fitzpatrick, & Norris, 2015), or disrupting species interactions that are important for natural regeneration (Fonturbel, Salazar, & Medel, 2017). Habitat loss also influences the carrying capacity of populations within communities. Populations falling below their carrying capacity may become susceptible to disease and threats from invasive species (Mora, Metzger, Rollo, & Ransom, 2017; Pimm, Raven, Peterson, Sekercioglu, & Ehrlich, 2006). These are just a few of the challenges that communities are currently facing, and coupled with climate change, the need to understand how anthropogenic disturbances will shape future communities is critical to conservation efforts (Brook, Navjot, & Bradshaw, 2008).

One of the key processes that will mediate these perturbations is dispersal (Liedvogel, Åkesson, & Bensch, 2011). Dispersal is the process by which populations and species redistribute in space and time. In its simplest form, dispersal can be any movement that provides potential for genetic mixing (Benton & Bowler, 2012; Ronce, 2007). Consequently, dispersal influences the genetic structure of populations in several ways. For example, less dispersive phenotypes, such as the flightless short‐winged morph of the cricket, *Gryllus rubens* may face greater risk of isolation as a result of habitat loss relative to the more dispersive fully winged flight‐capable morph (Mole & Zera, 1993). In fact, habitat persistence seems to maintain dispersal capability in wing‐dimorphic insect species (Denno, Roderick, Olmstead, & Döbel, 1991). Depending on the population genetic structure, less dispersive populations may face greater potential for inbreeding depression (Pimm et al., 2006). The negative impact from inbreeding depression can lead to the accumulation of deleterious alleles, particularly in small populations, and can ultimately lead to extirpation or extinction of species and whole communities (Mora et al., 2017). Similarly, and perhaps more importantly, dispersal represents one of the few routes for organisms to escape habitats that are under pressure from fragmentation (With & King, 1999). As such, the persistence of populations affected by habitat loss, including those that are genetically distinct, likely depend on individuals that can cross a matrix of hostile landscapes before reaching a suitable habitat, or use stepping‐stones in the habitat to connect with important resources (Watts et al., 2015).

**Figure 1:**
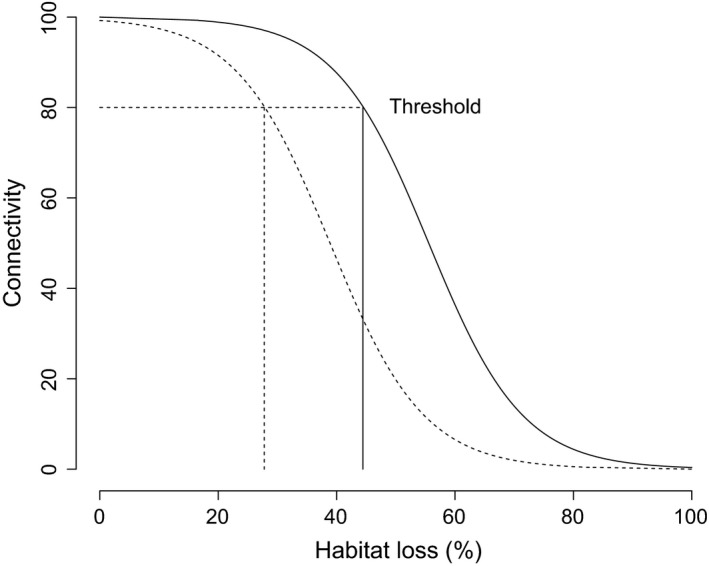
The theoretical nonlinear relationship between habitat loss (x‐axis), connectivity (y‐axis) and dispersal strategy (dotted and solid lines). Connectivity is defined as the chance of moving from one side of a landscape to the other. As the amount of habitat is removed connectivity declines steadily due to increasing fragmentation (e.g., increasing interpatch distances between habitat patches) until reaching a threshold (intercept of the horizontal and vertical lines) where connectivity decreases rapidly. The threshold is dynamic for a population with multiple dispersal strategies: for a dispersal‐limited strategy (broken line) connectivity begins to decrease rapidly already when less than 30% of habitat is removed (intercept of the two broken lines), while for a more dispersive strategy connectivity decreases rapidly when more than 40% of habitat is removed (intercept of the black and broken lines)

Habitat loss can reduce connectivity in the landscape by increasing the distance between suitable patches (Hanski, 1998). A few papers have illustrated this principle by modeling movement in landscapes as a function of habitat loss (Dytham & Travis, 2012; With & King, 1999). In these models, patches of habitat are usually removed from the landscape, and over time, the probability of moving from one side of the landscape to the other slowly decreases until reaching a *threshold* where dispersal is greatly reduced (Figure 1). Factors that influence these dispersal thresholds (e.g., the breaking point at which interpatch distance increases beyond dispersal propensity) are therefore fundamental, not only to understand the degree to which habitat loss impedes dispersal between patches, but also to understand how habitat loss may influence the evolution of dispersal strategy. Although researchers rarely discuss the dynamics of dispersal thresholds, it is logical to propose that the shape of the relationship between habitat loss and dispersal depends on the interaction between dispersal strategy and inter‐habitat distance. This generates a very interesting predicted nonlinear relationship between dispersal strategy and threshold distance, such that the distance at which dispersal will be significantly reduced is shorter for a dispersal‐limited strategy relative to a more dispersive strategy (Figure 1). Consequently, selection on dispersal strategy could result in changes in the position of the threshold.

Here, we empirically test this idea by assessing whether the shape of the relationship between habitat loss and dispersal depends on the interaction between interpatch distance and dispersal strategy. We test this using wild‐type strains of fruit flies (*Drosophila melanogaster*) that differ in dispersal strategy both in the laboratory and in nature (Edelsparre, Vesterberg, Lim, Anwari, & Fitzpatrick, 2014), and we expose them to environments with decreasing connectivity. In our experiments, there is no habitat loss per se; rather, we use increasing distances between patches to simulate the decreasing connectivity that arises from habitat loss (Hanski, 1998; With & King, 1999). Our prediction is that, in general, flies will show a nonlinear (e.g., threshold) decrease in dispersal in response to increasing interpatch distances and that the relationship between dispersal and interpatch distance will depend on dispersal strategy (Figure 1). To establish whether changes in connectivity can influence the evolution of dispersal strategies within populations, we assessed whether dispersal thresholds are associated with naturally occurring genetic variation in *foraging* (*for*), a gene known to influence food‐searching and dispersal behavior in fruit flies (Edelsparre et al., 2014; Sokolowski, 2001).

## 2 MATERIALS AND METHODS

### 2.1 Fly lines

To test whether fruit flies with different dispersal strategies exhibit differences in response to changes in habitat connectivity, we reared two allelic variants of the *foraging* (*for*) gene (rover‐ *for* ^R^, sitter‐ *for* ^s^) (de Belle & Sokolowski, 1989). In both laboratory and field trials, homozygous *for* ^s^ individuals exhibit a dispersal‐limited phenotype relative to homozygous *for* ^R^ individuals (more dispersive) (Edelsparre et al., 2014). Individuals homozygous for *for* ^s^ (sitters) are characterized by lower *for*‐mRNA expression levels and protein activity (PKG) relative to individuals with the *for* ^R^ allele (rovers) (Osborne et al., 1997). The *for* ^R^ and *for* ^s^ strains used here differ in their second pair of chromosomes where *for* resides, but share *for* ^R^‐derived third and X chromosomes to provide some control for genetic background (de Belle & Sokolowski, 1989; Kaun et al., 2007).

To test whether differences in behavior can be directly linked to variation in *for,* we reared a third fly strain, the *for* ^s2^ mutant. The *for* ^s2^ mutant is essentially a rover with a laboratory‐generated mutation in *for* resulting in sitter‐like *for‐*mRNA expression, PKG activity, and behavior (de Belle *et al*. 1993; Pereira & Sokolowski, 1993). Because the *for* ^s2^ mutation was induced on the *for* ^R^ genetic background, the mutation provides a direct test as to whether any differences in phenotype can be linked with allelic variation in *for*.

Strains were kept on a yeast–sugar–agar medium (40 ml) in 170 ml plastic bottles (VWR) with sponge tops on a 12/12‐hour light/dark cycle at 23 ± 2°C (standard conditions; see Edelsparre et al., 2014). Experimental flies were collected upon emergence from pupae and kept in 90 ml plastic vials under standard conditions until they were between 5 and 7 days old.

### 2.2 Dispersal assay

**Figure 2:**
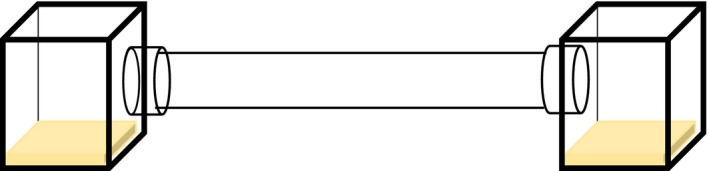
The two‐patch arena used to assay dispersal. Each cube represents a chamber (4.8 cm × 2.9 cm) filled with 2 ml standard yeast–sugar–agar medium (depicted in yellow). The horizontal lines drawn between the cubes represent the tubing which allowed flies to move unhindered between the two chambers. Three lengths of tubing were used in separate trials to simulate increasing distance arising from habitat loss: 20, 40, and 80 cm. All parts of the arena were made from clear see‐through materials to allow observers to monitor the movement of flies between the chambers

We quantified dispersal using a two food‐patch arena (Figure 2). This type of design has been used successfully for studies of connectivity in nature (Tewksbury et al., 2002). The arena consisted of two rectangular chambers each measuring 4.8 cm high and 2.9 cm wide (AMAC Plastic Products). The chambers were connected by tubing (Tygon, diameter 6.4 mm) to allow dispersal from one chamber to another. This procedure provided flies with a “familiar” environment and a choice between staying or dispersing into an unknown environment. The willingness to disperse into an unknown destination (i.e., the adjacent chamber) via the tube was assumed to reflect flies’ dispersal propensity. Each chamber was filled with 2 ml yeast–sugar–agar medium prior to each trial. An experimental trial involved gently transferring 16 CO_2_ anesthetized flies of the same sex and genotype and placing them into one of the chambers (designated as the start chamber) and allowing them 6 hr to disperse between chambers. Before an experimental trial commenced, we ensured that flies resumed normal behavior (e.g., male‐to‐male aggression, foraging, etc.) and that females had deposited eggs (see definition of dispersal above). The proportion of dispersers was quantified as the proportion of flies that had moved to the other chamber. Previous chamber‐to‐chamber dispersal assays on this system yielded rover/sitter differences in dispersal at distances of 4 cm over 6 hr (Edelsparre et al., 2014). This simple laboratory assay is an accurate predictor of rover/sitter dispersal in nature (Edelsparre et al., 2014). To simulate decreasing connectivity between the two patches, we exposed new flies to similar dispersal assays as described above, but we connected the two chambers with tubing lengths of 20 cm, 40 cm, and 80 cm, in separate trials. By doubling the distance at each level of connectivity, we attempted to identify a range of distances that might uncover thresholds between the two dispersal strategies while also being logistically feasible to execute in the laboratory along with balancing the number of replicates and treatments. In total, we conducted 204 dispersal trials (rover *n* = 65, sitter *n* = 67, sitter mutant *n* = 72). For all trials, the tubing length, arena orientation, and start chamber were randomized to control for sequential and directional effects.

### 2.3 Statistical analysis

To test whether changes in connectivity differed as a function of *for*‐mediated dispersal strategies, we used a two‐step approach. First, we tested whether the relationship between dispersal and interpatch distance differed between genotypes (genotype × distance interaction). To do this, we used a three‐way ANOVA fitting interpatch distance as the main effect and strain as a second effect. We included sex as a third effect to control for differences between males and females. Finally, to assess whether each strain exhibited dispersal thresholds, we calculated the 95% confidence interval around the mean proportion that reached the adjacent chamber at each distance. Distances at which the confidence interval within each strain did not overlap were taken as evidence for a threshold. To meet assumptions of normality proportions were arc‐sine square root transformed, however, for ease of interpretation, we plot the untransformed data to construct our figures. All analyses were conducted using R (v. 3.3.3; R Development Core Team, Vienna, Austria).

## 3 RESULTS

**Figure 3:**
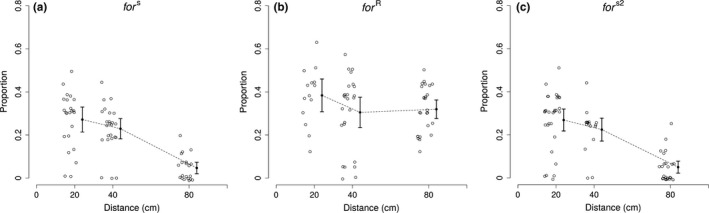
The proportion of flies dispersing to an adjacent food patch (*y*‐axis) at increasing distances (*x*‐axis). Each distance represents a degree of connectivity with highest connectivity at 20 cm between food patches and lowest connectivity at 80 cm. Open circles represent the proportion dispersing for a given trial, closed circles represent the mean proportion dispersing for each distance, and whiskers associated with each closed circle represent the 95% confidence intervals. The broken line trends the mean proportion dispersing across all distances. For flies with a dispersal‐limited strategy (*for* ^s^, sitters), the mean proportion of flies dispersing decreases with distance until reaching a break point (a) where connectivity decreases rapidly. For flies with a more dispersive strategy (*for* ^R^, rovers), the proportion of flies dispersing to the adjacent food patch is unaffected across all three distances, and there was no evidence for a break point in connectivity (b). The *for* ^s2^ mutation (c) induces a sitter‐like phenotype (e.g., dispersal‐limited strategy), resulting in a break point in connectivity that mirror sitter flies

There was a clear interaction between strain and interpatch distance on the proportion of dispersing flies (Strain × Distance: *F*_4,186_ = 6.28, *p = *.0001). In general, the proportion of flies dispersing decreased with interpatch distance (Distance: *F*_2,186_ = 29.99, *p *<* *.0001); however, this effect differed between the rover and sitter strains (Strain: *F*_2,186_ = 43.31, *p *<* *.0001); the proportion of dispersing sitter flies decreased with larger distances (Figure 3a) and this decrease was significantly greater when the interpatch distance was increased to 80 cm (Figure 3a), consistent with the notion of a threshold. Conversely, rovers dispersed in greater proportions than sitters across all distances and there was no evidence of a threshold in connectivity for rover flies across the three distances we tested (Figure 3b). In general, females dispersed in greater proportions across all three distances (Sex: *F*_1,186_ = 8.63, *p = *.0037), but this effect did not include rovers (strain × sex: *F*_4,186_ = 3.46, *p = *.0032). The differences observed between the rover and sitter strains can be directly attributed to genetic variation at *for*. When *for* ^s2^ flies were exposed to increasing interpatch distance, their dispersal pattern mirrored that of sitters (CI's overlap at all distances). Furthermore, the “position” of the threshold of both sitters and *for* ^s2^ flies was between 40 cm and 80 cm and did not significantly differ from each other (sitter CI = 0.02–0.07, *for* ^s2^ = 0.02–0.08; compare Figure 3a with Figure 3c). This suggested that the differential responses to changes in connectivity between rovers and sitters arose from variance in the *for* gene.

## 4 DISCUSSION

Our findings support the idea that the interaction between dispersal strategy and the spatial distance between habitat patches determines dispersal thresholds and therefore can influence the degree of connectivity in landscapes. This conclusion is reinforced by our finding that dispersal between two habitat patches decreased rapidly (threshold) with increasing distance in flies with a dispersal‐limited strategy, whereas no significant reduction in dispersal was observed in flies with a more dispersive strategy. As such, the degree of connectivity was reduced at significantly at shorter distances in genotypes with a dispersal‐limited strategy compared to a genotype with a more dispersive strategy. This is completely in line with our prediction.

The idea that genetic variation underlying dispersal strategy can be directly linked with the position of the threshold is also supported by our findings. The *for* ^s2^ mutation induced a sitter‐like dispersal phenotype. The proportion of *for* ^s2^ flies dispersing to the adjacent food patch decreased significantly at 80 cm, nearly identical to what we observed for flies with a natural dispersal‐limited strategy. This confirms that the difference in connectivity observed between rovers and sitters arose from variation in the *for* gene. This further suggests that decreasing connectivity arising from habitat loss could shift allelic frequencies in this system and consequently influence the evolution of dispersal strategy. During times of high connectivity, the two strategies may coexist; however, as more and more habitat is removed, the decrease in connectivity may select for increased dispersal and consequently shift the mean population dispersal threshold. In this system, one might predict that such a scenario would lead to an overall increase in *for* ^R^ alleles, but this remains to be tested.

Understanding factors influencing the degree of connectivity in landscapes is critical to implementing successful management of species and communities (van der Hoek, Zuckerberg, & Manne, 2015). Here, we show that interpatch distance interacts with dispersal strategy to determine thresholds at which connectivity breaks down. This idea may seem intuitive, however, most, if not all empirical examples focus on estimating the degree of connectivity in landscapes by considering the number and size of habitats in the landscape (van der Hoek et al., 2015; van Langevelde, 2000; Shultz & Crone, 2004). Our results suggest that researchers need to include distance when attempting to derive estimates of connectivity within a landscape. In particular, our results support the idea of Dytham and Travis (2012) wherein the scale (distance) and pattern (random vs. nonrandom) of loss interacts with dispersal to determine the degree of connectivity in the landscape. Including information of dispersal thresholds in determining connectivity could therefore be particularly valuable for constructing corridors between habitats, designing habitats with sufficient connectivity to sustain viable communities in human impacted landscapes and/or providing stepping‐stones for important gene flow between populations and communities of interest.

Our study further demonstrates that researchers need to consider intra‐population variation in dispersal strategy when examining the effect of habitat loss on connectivity. In our experiments, a decrease in connectivity had a more severe effect on sitter flies, whereas the dispersal tendencies of rovers remained unchanged across the three distances we tested. As such, habitat loss could lead to a reduction in genetic variation influencing dispersal or migratory behaviors (Betini et al., 2015; Liedvogel et al., 2011) if the average interpatch distance increases beyond the dispersal threshold of dispersal‐limited individuals. Similarly, constructing corridors with limited connectivity could impose selection for increased dispersal. Both scenarios could have detrimental effects if genetic variation underlying dispersal and migratory behaviors also act pleiotropically on functionally different traits. For example, *for* has pleiotropic effects on numerous traits, including larval foraging behavior (Sokolowski, 2001), aggression in adult males (Wang & Sokolowski, 2017), and oviposition site selection in females (McConnell & Fitzpatrick, 2017) (for extensive review and phenotypes, see Reaume & Sokolowski, 2009). Changes in connectivity could thus constrain or facilitate the evolution of all of these traits, not just dispersal. Experiments on the Eurasian blackcap, *Sylvia atricapilla*, demonstrated a genetic link between migratory traits as part of a migratory gene package (Berthold, 1999), and in fact most birds have this genetic ‘machinery’. Habitat loss could thus affect a suite of additive genes and influence the migratory pattern of many bird species.

We raise three potential caveats to our study. Firstly, we did not investigate changes in connectivity arising from habitat loss per se, rather we investigated the flies’ propensity to cross distances between two food patches. This approach potentially evaluates the scale of habitat loss, but not the pattern of loss. Habitat loss can be random, aggregated, or a mixture, and the outcome of each of these patterns on connectivity in the landscape are likely going to be very different (Dytham & Travis, 2012). Nevertheless, our findings clearly demonstrate that dispersal thresholds can arise in one part of a population if the interpatch distance is increased beyond their propensity to disperse. Secondly, by doubling the distance at each level of connectivity, we were unable to precisely identify the location of the threshold. Our results suggest that, in our laboratory system, a threshold for dispersal‐limited flies lies somewhere between 40 cm and 80 cm. Additional distances would likely increase our ability to resolve the threshold distance. For example, there are three possible outcomes for dispersal had we collected data at 60 cm (in between our 40–80 range): (1) Proportions are similar to those collected at 40 cm, suggesting dispersal declines rapidly (threshold) somewhere after 60 cm. (2) Proportions are similar to those collected at 80 cm, suggesting dispersal declines rapidly somewhere before 60 cm. (3) Assuming comparable variability to our empirical data, proportions that lie directly between those collected for 40 and 80 cm (i.e., means between 0.135 and 0.145) could conceivably lead to overlapping confidence intervals that support both linear and nonlinear (threshold) declines in dispersal. Finally, due to the size of our experimental arenas, our study limited movements to walking. However, the simple tube‐to‐tube assay accurately mirrors rover/sitter dispersal patterns in nature (Edelsparre et al., 2014). Furthermore, the goal of our study was not to estimate realized dispersal thresholds in nature nor the end‐points of dispersal; rather, we set out to demonstrate the importance of considering the interaction between interpatch distance and dispersal strategy when assessing the degree of connectivity in landscapes. To this end, we believe our findings show a novel and powerful example of this interaction, which validates theoretical models and informs our understanding of how habitat loss may affect population persistence and therefore community structure.

Two main conclusions emerge from our study. Firstly, our study is one of few to empirically demonstrate the importance of considering interpatch distance and dispersal strategy when estimating connectivity in landscapes. In landscape ecology, there has been a tendency to focus on patch structure when addressing how structural connectivity (i.e., distribution and pattern of patches) influences functional connectivity (i.e., dispersal; With & King, 1999). We anticipate that our findings will serve as a reminder of what is intuitively appealing; that dispersal will decline if the distance from one habitat to another exceeds the dispersal propensity of an organism. Although our experiments are simple compared with landscape studies, our findings nonetheless highlight the caveat of not considering the spatial component of the gaps arising from habitat loss. In fact, our study further suggests that there may be a critical point (i.e., threshold) where gap structure imposes a rapid decline in connectivity. We argue that including such information in landscape models may improve correlations between landscape indices and dispersal (Schumaker, 1996; but see Tishendorf & Fahrig, 2002). Secondly, our study provides unique insights into how naturally occurring genetic variation in dispersal strategies can be differentially influenced by habitat loss. Our findings demonstrated a clear link between allelic variation in *for* and response to decreasing connectivity. Therefore, these critical thresholds may not be fixed for a given population or species, rather they are at least partially dependent on the dispersal capabilities of the organism in question. In turn, changes in connectivity could have important implications on the evolution and maintenance of variation in dispersal strategies (Cornelius, Awade, Candia‐Gallardo, Sieving, & Metzger, 2017; Cote et al., 2017). Our findings are therefore a reminder of the potential evolutionary changes that may be imposed by habitat fragmentation on dispersal and migratory behaviors in particular and correlated behaviors in general.

## CONFLICT OF INTEREST

None declared.

## AUTHOR CONTRIBUTION

AHE and MJF conceived and designed the experiments. AS executed the experiments. AHE and MJF performed the analyses. AHE wrote the initial draft and MJF contributed substantially to revisions.

## DATA ACCESSIBILITY

Data available from the Dryad Digital Repository: https://doi.org/10.5061/dryad.2rd20f3.
